# Is there continued evidence for an association between abacavir usage and myocardial infarction risk in individuals with HIV? A cohort collaboration

**DOI:** 10.1186/s12916-016-0588-4

**Published:** 2016-03-31

**Authors:** Caroline A. Sabin, Peter Reiss, Lene Ryom, Andrew N. Phillips, Rainer Weber, Matthew Law, Eric Fontas, Amanda Mocroft, Stephane de Wit, Colette Smith, Francois Dabis, Antonella d’Arminio Monforte, Wafaa El-Sadr, Jens D. Lundgren, for the D:A:D Study Group

**Affiliations:** Research Department of Infection and Population Health, University College London (UCL), Royal Free Campus, London, UK; Academic Medical Center, Division of Infectious Diseases and Department of Global Health, University of Amsterdam, Amsterdam, The Netherlands; Denmark Centre for Health and Infectious Disease Research (CHIP), Department of Infectious Diseases, Section 2100, Rigshospitalet, University of Copenhagen, Copenhagen, Denmark; Division of Infectious Diseases and Hospital Epidemiology, University Hospital Zurich, University of Zurich, Zurich, Switzerland; The Kirby Institute, University of New South Wales (UNSW), Sydney, Australia; Department of Public Health, Nice University Hospital, Nice, France; Le Centre Hospitalier Universitaire (CHU) Saint-Pierre, Department of Infectious Diseases, Brussels, Belgium; Université Bordeaux Segalen, INSERM U897, Epidemiologie-Biostatistique, CHU de Bordeaux, Bordeaux, France; Dipartimento di Scienze della Salute, Clinica di Malattie Infectitive e Tropicali, Azienda Ospedaliera-Polo Universitario San Paolo, Milan, Italy; ICAP-Columbia University and Harlem Hospital, New York, NY USA

**Keywords:** Abacavir, Cardiovascular disease, Myocardial infarction, Risk, Channelling bias, Confounding

## Abstract

**Background:**

In March 2008, the D:A:D study published results demonstrating an increased risk of myocardial infarction (MI) for patients on abacavir (ABC). We describe changes to the use of ABC since this date, and investigate changes to the association between ABC and MI with subsequent follow-up.

**Methods:**

A total of 49,717 D:A:D participants were followed from study entry until the first of an MI, death, 1 February 2013 or 6 months after last visit. Associations between a person’s 10-year cardiovascular disease (CVD) risk and the likelihood of initiating or discontinuing ABC were assessed using multivariable logistic/Poisson regression. Poisson regression was used to assess the association between current ABC use and MI risk, adjusting for potential confounders, and a test of interaction was performed to assess whether the association had changed in the post-March 2008 period.

**Results:**

Use of ABC increased from 10 % of the cohort in 2000 to 20 % in 2008, before stabilising at 18–19 %. Increases in use pre-March 2008, and subsequent decreases, were greatest in those at moderate and high CVD risk. Post-March 2008, those on ABC at moderate/high CVD risk were more likely to discontinue ABC than those at low/unknown CVD risk, regardless of viral load (≤1,000 copies/ml: relative rate 1.49 [95 % confidence interval 1.34–1.65]; >1,000 copies/ml: 1.23 [1.02–1.48]); no such associations were seen pre-March 2008. There was some evidence that antiretroviral therapy (ART)-naïve persons at moderate/high CVD risk post-March 2008 were less likely to initiate ABC than those at low/unknown CVD risk (odds ratio 0.74 [0.48–1.13]). By 1 February 2013, 941 MI events had occurred in 367,559 person-years. Current ABC use was associated with a 98 % increase in MI rate (RR 1.98 [1.72–2.29]) with no difference in the pre- (1.97 [1.68–2.33]) or post- (1.97 [1.43–2.72]) March 2008 periods (interaction *P =* 0.74).

**Conclusions:**

Despite a reduction in the channelling of ABC for patients at higher CVD risk since 2008, we continue to observe an association between ABC use and MI risk. Whilst confounding cannot be fully ruled out, this further diminishes channelling bias as an explanation for our findings.

## Background

In March 2008, the Data Collection on Adverse Events of Anti-HIV Drugs (D:A:D) study presented findings at the 15th Conference on Retroviruses and Opportunistic Infections in Boston demonstrating a 90 % increase in the risk of myocardial infarction (MI) in HIV-positive individuals receiving antiretroviral therapy (ART) regimens that included abacavir (ABC) [1]; these findings were published in *The Lancet* in April 2008 [2]. Subsequent attempts by other studies to replicate these findings have been inconsistent, with some studies reporting a similar association [3–9] and others not [10–12]. Other studies have reported that a significant association in univariable analyses disappeared after adjustment for renal dysfunction or use of recreational drugs [13, 14]. Published meta-analyses on the topic have also been inconsistent [15, 16]. Studies have explored mechanisms that could explain this association, for example, those which suggest that an increased risk of MI in patients receiving ABC may be a result of the propensity of the drug to induce platelet hyperreactivity [17–19].

One of the limitations of observational studies is the potential for confounding to introduce bias in any comparison of outcomes among those receiving different ART drugs. In the case of ABC, confounding was of real concern, as the drug was traditionally preferentially prescribed to those at higher underlying risk of cardiovascular disease (CVD), where clinicians had avoided the use of other nucleoside reverse transcriptase inhibitors (NRTIs) that were known to have adverse lipid effects [20]. Thus, individuals receiving ABC before our findings were presented in 2008 were expected to have a higher underlying risk of CVD as a result of this ‘channelling bias’. The initial D:A:D analyses had adjusted for all factors that were thought to potentially confound any association between ABC use and MI risk, including age, sex, HIV mode of acquisition, ethnicity, calendar year, cohort, smoking status, family and personal history of CVD, body mass index (BMI), and exposure to other ART drugs [1, 2]. Subsequent analyses additionally included adjustment for renal dysfunction [21], a factor also known to be associated with a higher underlying CVD risk, with similar findings. In addition, we provided further arguments against confounding as an explanation, notably that the association with MI appeared to be reversible on discontinuation of the drug, and that there was no similar association with tenofovir (another NRTI where channelling might be expected to act in the same direction). Nevertheless, there remained concern that it was not possible to fully account for the channelling of high CVD risk patients onto ABC, meaning that residual confounding may have remained.

Since the findings in the D:A:D publication of 2008 of a possible elevated risk of MI when ABC is prescribed in persons with already elevated underlying CVD risk, there have been subsequent changes to the package insert for ABC, and several changes to international guidelines for use of the drug as part of ART treatment of HIV-positive individuals [22, 23]. As a result, it is likely that prescribing patterns of ABC have changed, particularly for those at higher risk of CVD [24], meaning that the channelling of patients with high CVD risk onto ABC is reduced, absent or even reversed. In this paper, we describe changes in the use of ABC among participants in the D:A:D study since publication of the findings in March 2008, and investigate whether the association between ABC and MI remains present in data collected after this time, when ABC would be less likely to be prescribed to those at high CVD risk than before 2008. In this way, our report describes an example of how the various aspects of science have contributed to a better understanding of a potential causal association after a safety signal was first identified.

## Methods

### Study design

The D:A:D study is an observational study of >49,000 HIV-1-positive patients from 11 cohorts from Europe, Australia and the United States [25]. All participants were under active follow-up in their cohorts at the time of enrolment in the D:A:D study. The primary study aim is to investigate associations between the use of ART drugs and the risk of CVD and other major disease events. Data are collected prospectively during routine clinic visits; the standardised dataset includes information on socio-demographic factors (including ethnicity which is captured as part of individual cohort data collection processes, where permitted), AIDS events and deaths, known risk factors for CVD, laboratory markers for monitoring HIV (including CD4 count and HIV RNA) and CVD, ART and treatments that influence CVD risk. Enrolment in the D:A:D study took place in three phases: enrolment cohort I (enrolment from 1999–2000); enrolment cohort II (added in 2004); and enrolment cohort III (added in 2009).

Information on all incident cases of MI are reported to the study coordinating centre via a study event form which captures detailed information about the event and related circumstances. Study personnel (at the coordinating centre and at local sites) receive extensive training in the identification of events and completion of the event form. Once the event form has been received, each event is validated (with dialogue between the coordinating centre and local site to clarify any discrepancies or queries) and coded using criteria applied in the WHO MONICA Project [26]. Validation and coding is performed blind to information on the patient’s ART status. Reported MIs are classified as definite, possible and unclassifiable; events with insufficient evidence to verify that the event was, indeed, an MI were rejected. Standardised criteria for classification included relevant symptoms, relevant increase and decline in cardiac enzymes, ischaemic changes in electrocardiographic (ECG) readings and, in cases of death, autopsy results, if available. A sample of all MI events further undergoes additional checks by an external independent cardiologist, and a sample of patient records are verified at source to ensure that events are not missed. Non-fatal MIs not associated with clinical symptoms were not considered as events.

### Ethics, consent and permissions

All participating cohorts followed local national guidelines/regulations regarding patient consent and/or ethical review.

### Statistical methods

The demographic and clinical characteristics of individuals in the D:A:D study under follow-up on 1 January each year were summarised, as was the proportion of follow-up time in each year that was contributed by individuals receiving ABC (overall and after stratification by the individual’s 10-year CVD risk). Each individual’s 10-year CVD risk was calculated using the Framingham equation and was classified as low (<10 %), moderate (10–20 %), high (>20 %) or unknown. Note that although the D:A:D study has also published a CVD risk score [27], this was not in widespread use over the period of interest and so we used the Framingham score for stratification.

To investigate changes in the initiation of ABC as part of first-line ART regimens, we identified all ART-naïve individuals who initiated ART for the first time during prospective follow-up. Logistic regression models were used to assess associations of the calendar period (pre-/post-1 March 2008) and 10-year CVD risk (low/unknown or moderate/high) with whether the initial ART regimen included ABC or not. Analyses included adjustment for the following potential confounders: gender, mode of HIV acquisition, ethnicity, age, BMI, family (first degree relative) or personal history of an MI, and the individual’s CD4 count at the time of initiation of ART. To test whether the association between CVD risk and initiation of ABC had changed in the post-2008 era, tests of interaction were performed between calendar period and 10-year CVD risk group. Stratified analyses were performed to obtain estimates of the odds ratio for initiation of ABC according to CVD risk in the two calendar periods separately.

To investigate changes in the likelihood of discontinuing ABC in the post-March 2008 period, patients receiving an ABC-containing regimen were followed from the time of initiation of that regimen until discontinuation of ABC, or the earliest of death, 1 February 2013 or 6 months after the individual’s last clinic visit (the censoring date for all analyses). Poisson regression was used to describe associations between the calendar period, CVD risk group and the rate of ABC discontinuation, with adjustment for potential confounders as described above. Interaction tests and stratified analyses were again performed to assess whether associations between CVD risk and ABC discontinuation had changed over time. To reflect the fact that factors predicting ABC discontinuation may differ in the context of a suppressed or non-suppressed viral load, analyses were performed separately for follow-up time spent with a suppressed/low (≤1,000 copies/ml) or non-suppressed (>1,000 copies/ml) viral load; the cut-off of 1,000 copies/ml reflects the fact that in many clinical situations, clinicians may address adherence concerns first in individuals with detectable but low viral loads, before considering changes in ART.

Finally, to investigate whether the reported association between current use of ABC and MI risk has changed in the post-March 2008 period, we performed a similar analysis to that reported in the 2008 paper [2]. Specifically, individuals were followed from study entry to the time of an MI, with censoring as described above. As in previous analyses of the dataset, each individual’s follow-up was split into a series of consecutive 1-month periods and his/her clinical, immunologic and virologic status at the start of each period was established. Poisson regression was then used to assess the association between current use of ABC (with a 6-month grace period to allow for recent discontinuation) and MI risk, adjusting for use of other ART drugs and known confounders (fixed covariates: gender, mode of HIV acquisition, ethnicity, participating clinical cohort; time-updated covariates: age, smoking status, family history of CVD, previous CVD event, BMI, cumulative exposure to the main protease inhibitors and non-NRTIs, and cumulative and current exposure to other NRTIs). These analyses did not adjust for factors thought to potentially lie on the causal pathway between ABC use and MI risk, due to the possibility of the introduction of bias to such analyses. Of note, reflecting our previous findings and the state of evidence surrounding the possible mechanism of any association with ABC, which would be consistent with a short-lived on/off effect, only current exposure to ABC was considered in analyses. Statistical tests of interaction were again performed to test whether the association between ABC and MI rate had changed in the post-March 2008 period. Subsequent sensitivity analyses additionally adjusted for factors that might potentially fall on the causal pathway between use of ABC and MI risk, including diabetes mellitus, total and high-density lipoprotein cholesterol (HDL-C), triglycerides, systolic and diastolic blood pressure, receipt of anti-hypertensive drugs or ACE inhibitors, blood glucose, Framingham score, weight loss or gain, and renal function (expressed using the latest creatinine measurement, categorised into eight equally sized groups with a further category for those with missing creatinine measurements), all as time-updated covariates. Further sensitivity analyses incorporated the latest creatinine measurement in its continuous form, and also expressed renal function using the estimated glomerular filtration rate (either as a categorical or continuous covariate).

All analyses were performed using SAS version 9.13 (SAS Institute, Cary, NC, USA).

## Results

### Changes in the use of ABC over time

The D:A:D cohort currently comprises 49,717 individuals, of whom 73.8 % are male. Median age at cohort entry was 38 (interquartile range 32–44) years. The majority of participants were infected with HIV through sex between men (44.1 %) or sex between men and women (32.6 %), with 15.3 % infected via injection drug use and 8.0 % through other or unknown routes. Around half of the cohort (50.6 %) is white, with 6.8 % of black African origin and 2.9 % of other ethnicities; information on ethnicity is unknown for 36.7 % of the cohort, largely because the collection of information on ethnicity is prohibited in several participating countries.

By 1 February 2013, individuals in the cohort had accrued a total of 367,559 person-years (PYRS) of follow-up (median 7.0, interquartile range 4.4–11.1 years), 210,250 of which had been accrued before 1 March 2008 and 157,309 after 1 March 2008. The changing characteristics of the cohort over time are shown in Table 1; as expected, there has been a gradual increase in the age of the cohort over time and a gradual increase in the proportion with an AIDS event. Among those with known smoking status, the proportion of current smokers has dropped somewhat since 2005. Despite the ageing of the cohort, there have been positive changes in the proportion with dyslipidaemia and, in line with increased use of effective combination ART (cART), increases in the median CD4 count and decreases in the median HIV RNA level over time.

**Table 1:** Demographic and cardiovascular risk characteristics of the D:A:D cohort over time. The information shown in the table describes the current status of each individual under follow-up on 1 January each year (based on the last available measurement for that individual prior to the start of the year)

		Individuals under follow-up on 1 January of year:												
		2000	2001	2002	2003	2004	2005	2006	2007	2008	2009	2010	2011	2012
*N*		3,508	20,868	22,816	25,694	27,148	28,526	29,570	30,549	31,832	35,146	34,438	32,662	31,112
Male (%)		79.8	75.2	75.5	74.7	74.1	73.3	73.0	72.8	73.1	73.2	73.5	73.5	73.6
Age (years)	Median	39	39	40	41	42	43	44	45	46	47	48	49	50
Previous AIDS (%)		25.9	25.8	26.3	26.3	26.6	26.7	26.8	27.1	26.9	26.5	26.9	27.5	27.8
10-year CVD risk (%)	Low	5.1	35.7	42.3	45.4	49.5	52.8	57.0	61.9	64.2	63.9	66.8	69.8	71.7
	Moderate	1.7	7.8	9.1	8.8	9.1	9.3	9.1	9.2	9.9	10.5	10.7	11.0	11.3
	High	2.8	4.6	5.3	5.1	5.2	5.1	4.8	4.9	5.3	5.4	5.6	5.8	6.0
	Unknown	90.7	51.9	43.4	40.7	36.2	32.8	29.2	24.0	20.7	20.2	16.9	13.4	11.1
Smoking status^a^	Current smoker	27.2	49.2	49.6	49.0	47.2	47.5	45.5	45.0	44.1	43.8	42.7	41.0	39.8
	Ex-smoker	50.6	21.4	21.8	20.8	22.2	22.4	24.7	25.3	26.1	26.1	27.5	29.3	30.6
	Never smoked	22.3	29.4	28.6	30.2	30.6	30.0	29.8	29.7	29.8	30.1	29.9	29.7	29.6
Family history of CVD (%)		5.1	8.7	8.7	8.3	8.2	8.1	8.0	8.0	7.7	7.3	7.5	7.7	7.8
Diabetes (%)		3.6	3.9	4.5	4.7	5.1	5.0	5.0	5.0	5.1	5.2	5.6	5.9	6.3
TC (mmol/L)	Median	5.1	5.1	5.1	5.0	5.0	4.9	4.9	4.8	4.8	4.9	4.9	4.9	5.0
HDL-C (mmol/L)	Median	1.1	1.1	1.1	1.2	1.2	1.2	1.2	1.2	1.2	1.2	1.2	1.2	1.2
TG (mmol/L)	Median	1.9	1.7	1.7	1.7	1.7	1.6	1.6	1.5	1.5	1.5	1.5	1.5	1.5
CD4 (cells/mm^3^)	Median	380	448	461	465	460	460	468	480	494	503	527	548	566
Viral load (log_10_ copies/ml)	Median	2.4	2.1	1.9	1.8	1.7	1.7	1.7	1.7	1.7	1.7	1.7	1.7	1.7

^a^Expressed as a proportion of those with known smoking status, which has increased from 71.9 % of the cohort in 2000 to 89.5 % in 2012. *CVD* cardiovascular disease, *HDL-C* high-density lipoprotein cholesterol, *TC* total cholesterol, *TG* triglycerides

**Fig. 1:**
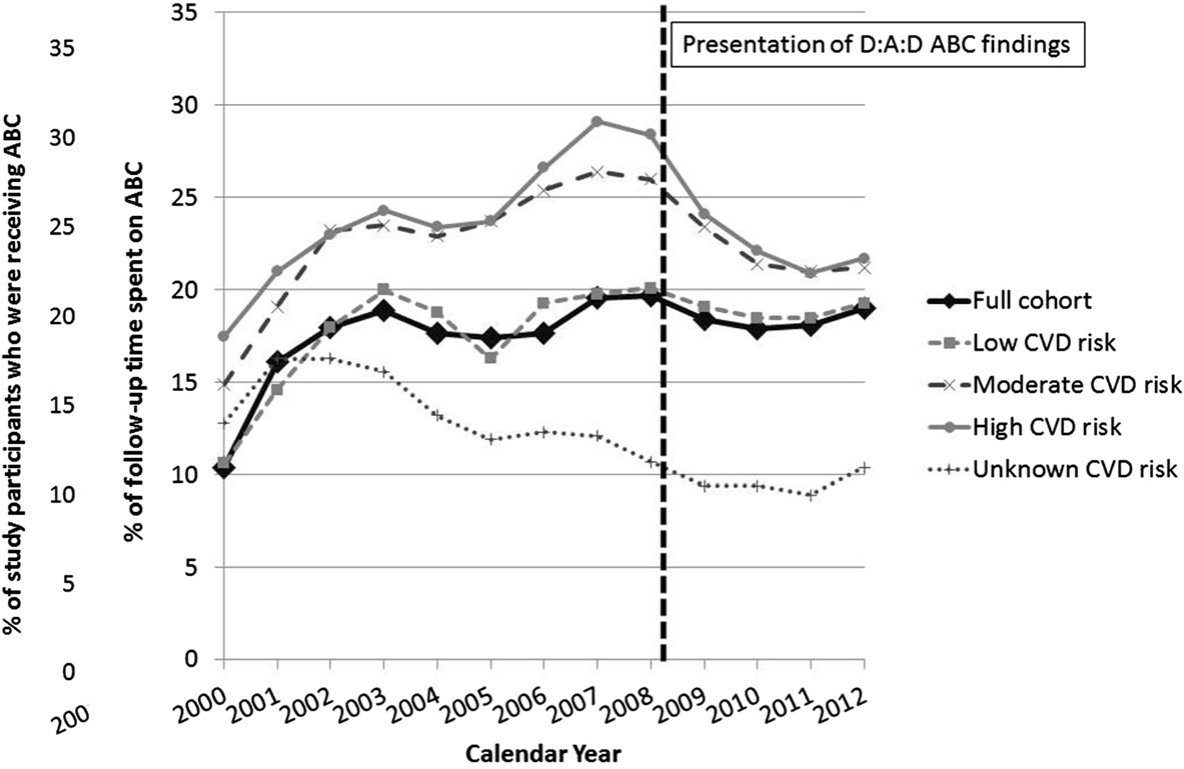
Changes to the use of ABC in the D:A:D cohort over time, overall and among groups stratified by CVD risk

Use of ABC among cohort participants increased from 10.4 % of those under follow-up in 2000 to 19.7 % of those under follow-up in 2008. Since then, the rate has stabilised at around 18–19 % (Fig. 1). After stratification by 10-year CVD risk, increases in use of ABC pre-March 2008, and subsequent decreases, were seen to be greatest in those at moderate and high CVD risk. For example, among those at moderate CVD risk in each year, use of ABC increased from 14.9 % in 2000 to 25.4 % in 2008 before dropping 21.2 % in 2012. Similarly, among those at high CVD risk, use of ABC was 17.5 %, 26.6 % and 21.7 % in these three years, respectively.

#### Treatment initiations (Table 2i)

**Table 2:** Association between 10-year CVD risk and i) initiations or ii) discontinuations of ABC in the pre- and post-March 2008 periods (factors included in adjusted analyses are listed in the Methods section)

Framingham risk group	ABC initiations/total ART initiations	Percentage (95 % CI)	aOR (95 % CI)
i) Initiations of ABC			
Pre-March 2008			
Low/unknown	1,251/9,213	13.6 (12.8, 14.3)	1
Moderate/high	111/648	17.1 (13.9, 20.3)	1.14 (0.90, 1.44)
Post-March 2008			
Low/unknown	326/4,282	7.6 (6.8, 8.4)	1
Moderate/high	33/622	5.3 (3.5, 7.1)	0.74 (0.48, 1.13)
Interaction *P* value			0.007
ii) Discontinuations of ABC	Discontinuations/PYRS	Rate/100 PYRS (95 % CI)	aRR (95 % CI)
Suppressed/low viral load			
Pre-March 2008			
Low/unknown	2,045/16,506	12.4 (11.9, 12.9)	1
Moderate/high	562/5,465	10.3 (9.4, 11.1)	1.04 (0.93, 1.16)
Post-March 2008			
Low/unknown	1,403/13,950	10.1 (9.5, 10.6)	1
Moderate/high	880/6,560	13.4 (12.5, 14.3)	1.49 (1.34, 1.65)
Interaction *P* value			0.0001
Non-suppressed viral load			
Pre-March 2008			
Low/unknown	2,966/7,766	38.2 (36.8, 39.6)	1
Moderate/high	662/2,041	32.4 (29.9, 34.9)	0.99 (0.90, 1.09)
Post-March 2008			
Low/unknown	622/2,297	27.1 (25.0, 29.2)	1
Moderate/high	236/921	25.6 (22.4, 28.9)	1.23 (1.02, 1.48)
Interaction *P* value			0.07

*ABC* abacavir, *aOR* adjusted odds ratio, *aRR* adjusted rate ratio, *ART* antiretroviral therapy, *CI* confidence interval, *CVD* cardiovascular disease, *PYRS* person-years

Overall, there were 14,765 new initiations of ART among ART-naïve patients, of which 1,721 (11.7 %) involved initiation of ABC. Among the 9,861 ART initiations that occurred prior to March 2008, 1,362 (13.8 %) involved ABC, compared to 359 of 4,904 (7.3 %) ART initiations occurring after March 2008. In the pre-March 2008 era, those with a moderate or high CVD risk were somewhat more likely to initiate ABC as part of first-line regimens than those with low or unknown CVD risk (adjusted odds ratio 1.14 [95 % confidence interval (CI) 0.90–1.44]). In contrast, in the post-March 2008 period, those with moderate or high CVD risk were less likely to initiate first-line regimens that included ABC (0.74 [0.48–1.13]). A statistical interaction test demonstrated that this change was statistically significant (*P* = 0.0007).

#### Treatment discontinuations (Table 2ii)

Among those with a suppressed viral load, there were 4,890 ABC discontinuations over a total follow-up of 42,481 PYRS (rate 11.5/100 PYRS). Prior to March 2008 there had been a total of 2,607 ABC discontinuations over 21,971 PYRS (11.9/100 PYRS) and this dropped to 2,283 discontinuations over 20,510 PYRS (11.1/100 PYRS) post-March 2008. These patterns differed, however, by 10-year CVD risk score, with a decrease in discontinuation rate from 12.4 to 10.1/100 PYRS in those with low or unknown CVD risk score, but an increase in discontinuation rates from 10.3 to 13.4/100 PYRS in those with moderate or high CVD risk. Compared to those with a low or unknown CVD risk, those with a moderate or high CVD risk had a similar rate of switching in the pre-2008 era (adjusted rate ratio (aRR) 1.04 [0.93–1.16]) but in the post-March 2008 era those at moderate or high CVD risk were significantly more likely to discontinue ABC (aRR 1.49 [1.34–1.65], *P* value for interaction = 0.0001).

Among those with a non-suppressed viral load, there were 4,486 ABC discontinuations over a total follow-up of 13,025 PYRS (rate 33.4/100 PYRS). Prior to March 2008 there had been a total of 3,628 ABC discontinuations over 9,808 PYRS (37.0/100 PYRS) and this dropped to 858 discontinuations over 3,217 PYRS (26.7/100 PYRS) post-March 2008. Whilst overall there was a decrease in discontinuation rates over the two periods, regardless of the individual’s underlying CVD risk score, the relative decrease in discontinuation was lower in those with a moderate or high underlying risk. Thus, whereas in the pre-March 2008 era, the results suggested that those with low/unknown CVD risk had a similar discontinuation rate to those with moderate or high CVD risk (aRR 0.99 [0.90–1.09]), in the post-March 2008 era, those with moderate or high CVD risk were 23 % more likely to discontinue ABC than those with low or unknown risk (aRR 1.23 [1.02–1.48], *P* value for interaction = 0.07).

### Association between current use of ABC and MI risk

By 1 February 2013, 941 MI events had occurred in the cohort (rate 0.26 [95 % CI 0.24–0.27]/100 PYRS). Overall, the rate of MI was 0.47 [0.42–0.52]/100 PYRS among those currently receiving ABC and 0.21 [0.19–0.22]/100 PYRS among those not currently receiving ABC. After adjustment for potential confounders, current ABC use was associated with a 98 % increase in MI rate (aRR 1.98 [1.72–2.29]), with no difference in the pre- (1.97 [1.68–2.33]) and post- (1.97 [1.43–2.72]) March 2008 periods (*P* value for interaction = 0.74) (Table 3).

**Table 3:** Results from multivariable Poisson regression models of association between current receipt of ABC and MI rate, overall and stratified by calendar period. Factors included in adjusted analyses are listed in the Methods section

	MI/PYRS	Rate (95 % CI)/100 PYRS	aRR (95 % CI)
Overall			
Not currently on ABC	600/295,642	0.20 (0.19, 0.22)	1
Currently on ABC	341/71,917	0.47 (0.42, 0.52)	1.98 (1.72, 2.29)
Pre-March 2008			
Not currently on ABC	425/169,417	0.25 (0.23, 0.28)	1
Currently on ABC	247/40,833	0.61 (0.53, 0.68)	1.97 (1.68, 2.33)
Post-March 2008			
Not currently on ABC	175/126,225	0.14 (0.12, 0.16)	1
Currently on ABC	94/31,084	0.30 (0.24, 0.36)	1.97 (1.43, 2.72)
Interaction *P* value			0.74

*ABC* abacavir, *aRR* adjusted rate ratio, *CI* confidence interval, *MI* myocardial infarction, *PYRS* person-years

#### Sensitivity analyses

Results were unchanged after stratifying by Framingham risk group, or after further adjusting for factors potentially on the causal pathway, including renal function, dyslipidaemia and hypertension (Table 4).

**Table 4:** Results from sensitivity analyses. i) Adjusted rate ratio for the association between MI and ABC, stratified by calendar period and CVD risk, and ii) adjusted rate ratio for the association between MI and ABC after further adjustment for factors that potentially lie on the causal pathway (see Methods section)

	Pre-March 2008		Post-March 2008		*P* value (interaction between ABC use and calendar period)
Sensitivity analysis	MI event rate (95 % CI)/100 PYRS	aRR (95 % CI)	MI event rate (95 % CI)/100 PYRS	aRR (95 % CI)	
i) Stratified by Framingham risk					
Low	0.14 (0.12, 0.16)	2.48 (1.74, 3.52)	0.08 (0.06, 0.09)	2.11 (1.12, 3.98)	0.78
Moderate	0.69 (0.59, 0.80)	1.69 (1.21, 2.35)	0.32 (0.26, 0.39)	2.19 (1.25, 3.84)	0.68
High	1.80 (1.56, 2.05)	1.51(1.12, 2.05)	0.74 (0.58, 0.90)	1.65 (0.93, 2.92)	0.16
ii) Further adjustment for factors on causal pathway	n/a	1.88 (1.55, 2.28)	n/a	2.03 (1.46, 2.84)	0.61

*ABC* abacavir, *aRR* adjusted rate ratio, *CI* confidence interval, *CVD* cardiovascular disease, *MI* myocardial infarction

## Discussion

It is clear that there has been some reduction in the use of ABC among those at higher risk of CVD in the more recent period. In particular, since March 2008 individuals at moderate or high CVD risk have been somewhat less likely to initiate ABC as part of first-line ART regimens, and more likely to discontinue ABC compared to their counterparts with low or unknown underlying CVD risk. Despite this reduction in the use of ABC in higher risk persons, we continue to observe a strong association between current ABC use and MI risk. This association remains unchanged, even after adjustment for the majority of known risk factors for CVD that may have influenced the choice of NRTI within this population. While it is not possible to adjust for unmeasured confounders, the reduction in the use of ABC among individuals with high measured CVD risk is likely also to have occurred in individuals with observed but unmeasured high CVD risk. This strongly suggests that our finding of a raised risk of MI for people on ABC is not explained by channelling of people with high CVD risk onto the drug. These results are of particular importance at a time when the use of ABC, as part of a new single tablet regimen, is likely to increase within many settings. The clinical relevance of our findings will always be dependent on the underlying risk of the event in a given individual. However, the relative rate that we see is similar even in those at high CVD risk, raising particular concerns about the use of ABC in this group.

Since the original publication from the D:A:D Study Group in 2008 [2], several research groups have attempted to replicate the findings from the study; the findings from these studies have been reviewed in detail in several recent review articles [28, 29]. The strongest associations with ABC have, arguably, come from observational cohort studies. In 2008, the combined SMART/INSIGHT and D:A:D study groups reported findings based on the subgroup of patients who had been randomised to receive continuous ART in the SMART trial [3]; current use of ABC was associated with an excess risk of clinical MI and major CVD events, both when a strict and expanded definition for CVD was considered. Three studies from the US Department of Veterans Affairs (VA) Clinical Case Registries investigated the association. In the study from Bedimo et al. [14], ABC-containing cART was associated with a 27 % increase in the risk of MI; this association was reduced, although remained raised, after controlling for markers of chronic kidney disease (adjusted rate ratio 1.23) and for known risk factors for MI (1.18). In an updated analysis of the same dataset [7], use of ABC was significantly associated with several outcomes, including atherosclerotic CVD events, coronary disease and cerebrovascular disease, with non-significant raised risk also seen for heart failure. Finally, Desai et al. reported a significant odds ratio of 1.50 for current abacavir exposure in a study published in 2015 [30]. The risk of hospitalisation with MI among participants in the Danish HIV cohort study [5] was doubled in ABC users compared with non-ABC users. In contrast, no association was seen between MI risk and ABC use in 6,517 patients receiving HIV care at one of two hospitals in Boston [10]. More recently, Marcus et al. [31] reported that ABC users in the Kaiser Permanente Northern and Southern California cohort had a 2.2-fold higher risk of CVD compared with patients initiating regimens without ABC.

Several case-control studies have been conducted to investigate the association. In the French Hospital’s Database on HIV [13], 289 individuals with a first definite/probable MI occurring between 2000 and 2006 were each matched to up to five control individuals with no history of MI. Whilst recent and past ABC use in this study was more likely to be seen in cases than in controls, these associations were marginally non-significant. In Montreal [6], Durand et al. reported that increased recent exposure to ABC, didanosine (ddI), stavudine (d4T) and zalcitabine (ddC) was found in cases with MI compared to controls without MI.

One of the limitations of observational studies is the potential presence of confounding. As patients are not randomised to the choice of therapy, the characteristics of those starting different drugs are likely to differ, and this may introduce bias if these characteristics are also associated with CVD risk. Due to the randomisation process employed, randomised controlled trials (RCTs) are generally thought to be free of confounding and, as such, provide a high-level of evidence to support a causal association. In the STEAL trial [4], serious non-AIDS events (including CVD) and ischaemic CVD both occurred significantly less frequently in individuals randomised to tenofovir/emtricitabine compared to those randomised to ABC/lamivudine. The trial was, however, a small one and had not been powered to detect a difference in these secondary endpoints.

Several meta-analyses of randomised trials have also been performed [11, 12, 15, 16] and have failed to demonstrate an association between ABC use and MI risk. Results from these meta-analyses must be interpreted, however, in the context of a population of largely ART-naïve individuals who are generally healthier and at lower CVD risk (due to trial inclusion/exclusion criteria, particularly relating to age restrictions and the presence of co-morbidities) than the wider HIV population. Many of the trials included in these meta-analyses were conducted at a time when associations with CVD had not yet been recognised, and MI and CVD were not captured as pre-specified endpoints. The under-ascertainment of events that may result, combined with an already low underlying event rate, is likely to contribute to the lack of associations seen in these meta-analyses. Of note, a recent study that attempted to emulate a RCT using data from a North Carolina Medicaid dataset, did report an increased rate of MI among patients initiating ABC compared with tenofovir [32]. A recent meta-analysis of observational studies [29] also reported an association between use of ABC and MI risk, although the pooled estimates were based on only the D:A:D [2] and Danish [5] studies.

One of the reasons for the continued debate about the potential association between ABC and MI is that there is no confirmed biological mechanism for the association. The MI signal associated with use of ABC is maintained as long as the drug is continued (i.e. over many years), eliminating the possibility that this is an adverse effect related to the well-known hypersensitivity reaction that may present early after drug initiation in persons with a HLA-B*5701 haplotype. Nevertheless, as the mechanism for the hypersensitivity reaction develops when the drug is presented by the HLA system to T-lymphocytes, it is possible that other HLA haplotypes may also convey a similar risk but without a distinct clinical phenotype. This possibility was supported by early cross-sectional analyses of the SMART trial [3], which suggested that baseline levels of high sensitivity C-reactive protein (hs-CRP) and IL-6 at baseline were higher in those receiving ABC than in those receiving other NRTIs. Subsequent analyses of follow-up information from the STEAL study [33] and other (generally small) studies [34–36] provided an inconsistent picture, with associations reported in some studies [37–39] but not others [40–45]. Recently, in vitro and in vivo animal experiments have demonstrated the ability of ABC to induce inflammation of the vascular wall by enhancing leucocyte attachment to endothelial cells through enhanced expression and/or function of the macrophage-1 antigen [46–48]. A recent study from Papakonstantinou et al. [49] demonstrated an increased level of platelet activating factor (PAF), a potent inflammatory factor, at 3 months after individuals started ABC suggesting a link between ABC use and MI risk. The possibility of a different underlying mechanism for the development of atherosclerosis and CVD in treated and untreated individuals [50] may explain differences in outcomes between studies of ART- naïve individuals starting cART for the first time (as is commonly the case in randomised trials), and those who are on established treatment (as is more often the case in observational studies).

ABC use has been shown to impair endothelial function [39]. Unfortunately, no measurements of endothelial function are currently available from randomised trials that compare virologically suppressive regimens of ABC versus non-ABC-containing regimens. The mechanisms that explain this observation also remain to be elucidated, but the studies that suggest that ABC causes vascular endothelial inflammation may hint at one possibility [46–48]. Conversely, ABC is not known to affect lipid or glucose metabolism nor accelerate subclinical atherosclerosis. This is in contrast to the HIV protease inhibitors, which likely accelerate CVD risk by perturbing lipid metabolism generally and, in particular, in macrophages involved in plaque formation.

Arguably, the most promising potential mechanism to date relates to the ability of ABC to lead to platelet reactivity [17, 18]. The metabolite of ABC, carbovir triphosphate, is a competitive inhibitor of the guanylyl cyclase enzyme in platelets [18], and this inhibition lowers the cyclic guanosine monophosphate (cGMP) concentration resulting in platelets that are more reactive. An association with hyperreactive platelets, with their short lifespan, would be consistent with the reversible nature of the ABC association with MI, and with the findings related to increased expression of PAF in the first few months on ABC [49, 51]. In support of this, Falcinelli et al. [19] reported findings suggesting that treatment with ABC enhances in vivo platelet activation and induces platelet hyperreactivity by blunting the inhibitory effects of nitric oxide on platelets. Seemingly in contrast to these findings, a recent Danish study [52] found evidence of reduced platelet aggregation and clot initiation in both treated and untreated HIV-positive individuals. However, the authors did not state how many patients in the treated group were receiving ABC, nor did they consider any specific drug associations.

Clearly, confounding cannot be ruled out in any cohort study. Although our initial report provided several arguments against confounding as an explanation, this concern continued to be levelled against the study. In particular, there has been a frequently raised concern that channelling by renal function (whereby those with poorer renal function would be preferentially placed on ABC-containing regimens to avoid use of tenofovir, a drug that has well-known renal toxicities) might induce an artificial association with MI, due to the known correlation between renal function and CVD risk. This was first raised by Bedimo and colleagues [14] who reported that after adjustment for renal function the *P* value for the association between MI and ABC use changed from 0.06 to 0.11. However, it is noteworthy that the hazard ratio itself was reduced only slightly from 1.27 to 1.23, indicative of only a modest confounding effect of renal function. The later study from the VA group did include adjustment for renal function, both at baseline and over follow-up [7] with no attenuation of association; Marcus et al. [31] also confirmed that their association was independent of renal function. Further, in an updated analysis from the D:A:D group [21] (as well as in the present analysis), the reported association with ABC was unchanged after adjustment for various measures of renal function. Confounding by recreational drug use has also been proposed as a possible explanation for the findings [13], although the VA analysis [7] did also include adjustment for this factor and clinicians within the D:A:D study reported that this information, if available, would be unlikely to influence choice of cART. Most importantly, we consider that, the fact that the reported association between MI and ABC is unchanged, provides the strongest evidence against channelling bias as an explanation for our findings.

One concern in analyses such as this is the possibility of time-varying confounding. If factors that are associated with the initiation of ABC also lie on the causal pathway between the use of abacavir and MI risk, then adjustment for these factors in standard multivariable analyses will generally result in biased estimates of the relative risk. For this reason, we did not include such factors in our primary analysis. However, the fact that the relative risk associated with current abacavir use was virtually unchanged in our sensitivity analyses which did include factors thought to be potentially on the causal pathway suggests that little time-varying confounding exists with these factors. This is also supported by the mechanistic studies which suggest a very different pathway for the association seen.

## Conclusion

In conclusion, there has been some channelling of ABC away from those at higher risk of CVD since 2008 when this safety signal was first identified. Despite this, we continue to observe a strong association between current ABC use and MI risk. The signal is furthermore supported by some, but not all, other observational studies, whereas meta-analyses of previous RCTs of ABC do not support an association among low CVD risk populations. Outside of the HIV field there have been well-documented examples of reported safety signals from observational studies that have failed to be confirmed in large-scale randomised trials (for example, [53]). However, in these cases, there is rarely an evidence-based biological mechanism for the associations seen. The clinical relevance of the ABC signal with CVD has been explored by basic science studies that suggest several potential biological mechanisms to explain the association. Despite these advances, a suitably powered RCT able to provide definitive evidence on the safety of ABC in patients with elevated underlying CVD risk has not been undertaken and it is questionable, given this further analysis of the D:A:D study, whether such a trial would be deemed to be ethical. Unfortunately, the inconsistent findings from the studies, together with few attempts to generate evidence of better quality, leaves patients with HIV and their treating physicians with continued ambiguity as to the safety of using ABC in patients with elevated risk of CVD. We call for action to address this ambiguity, with a greater focus on mechanistic studies which may lead to greater insight into such an association.

### Availability of data and materials

The D:A:D Steering Committee encourages the submission of concepts for research projects. Concepts can be submitted for review by the D:A:D Steering Committee using an online form (http://www.cphiv.dk/Ongoing-Studies/DAD/Submit-research-concept). The research concept will undergo review by the D:A:D Steering Committee for evaluation of the scientific relevance, relevance to the D:A:D study, design, statistical power and feasibility. Upon completion of the review, feedback will be provided to the proposer(s). In some circumstances, a revision of the concept may be requested. If the concept is approved for implementation, a writing group will be established consisting of the proposers (up to three persons that were centrally involved in the development of the concept) and members of the D:A:D Steering Committee (or other appointed cohort representatives), statistical department and coordinating centre. All persons involved in the process of reviewing these research concepts are bound by confidentiality.
